# The *CARD9* Polymorphisms rs4077515, rs10870077 and rs10781499 Are Uncoupled from Susceptibility to and Severity of Pulmonary Tuberculosis

**DOI:** 10.1371/journal.pone.0163662

**Published:** 2016-09-29

**Authors:** Ioana Streata, January Weiner, Marco Iannaconne, Gayle McEwen, Marius Sorin Ciontea, Marian Olaru, Rosanna Capparelli, Mihai Ioana, Stefan H. E. Kaufmann, Anca Dorhoi

**Affiliations:** 1 University of Medicine and Pharmacy of Craiova, Human Genomics Laboratory, 200638 Craiova, Romania; 2 Max Planck Institute for Infection Biology, 10117 Berlin, Germany; 3 University of Naples Federico II, Department of Agriculture, 80055 Naples, Italy; 4 “Tudor Vladimirescu” Pneumophtisiology Hospital Runcu, 217390 Gorj, Romania; Rutgers Biomedical and Health Sciences, UNITED STATES

## Abstract

Genetic variants in the *CARD9* gene predispose to inflammatory disorders and chronic infectious diseases. Tuberculosis (TB), a chronic infectious disease affecting the lung, is lethal in *Card9*-deficient mice. We hypothesized that polymorphisms in the *CARD9* gene influence TB progression and disease-associated lung damage in humans. We tested genotype distributions of the *CARD9* polymorphisms rs4077515, rs10781499 and rs10870077 in TB patients and healthy subjects in a Caucasian cohort. SNPs were in linkage disequilibrium and none of the haplotypes was significantly enriched in the TB group. We determined total and differential leukocyte count, erythrocyte sedimentation rate and plasma abundance of cytokines and chemokines as markers for systemic inflammation and scored chest X-rays to assess lung involvement in TB subjects. Most disease parameters segregated independently of the *CARD9* haplotypes. In contrast to multifactorial chronic inflammation, selected genetic variants in the *CARD9* gene leave host responses apparently unaffected in TB, at least in the population analyzed here.

## Introduction

Caspase recruitment domain-containing protein 9 (CARD9) is a cytosolic adaptor abundant in myeloid cells which is essential for recognition of microbes [1, 2]. In experimental animals CARD9 protects against infection with the opportunistic fungus *Candida albicans* [1] and intracellular bacteria, such as *Listeria monocytogenes* [3], and is essential for control of tuberculosis (TB)[4]. During infection with the etiological agent of TB, *Mycobacterium tuberculosis* (Mtb), the adaptor integrates signals from various pattern recognition receptors [4, 5] and fine-tunes concentrations of pro-inflammatory (TNF-α; IL-6; IL-1) and regulatory (IL-10) cytokines. In line with its general propensity to regulate abundance of various TNF and IL-1 family members [6, 7], selected *CARD9* gene polymorphisms predispose to inflammatory diseases in humans [8–10]. Polymorphisms in *CARD9* have thus far not been studied in the context of human TB, a contagious disease causing extensive morbidity and mortality worldwide [11], with complex genetic components [12]. We investigated several *CARD9* single nucleotide polymorphisms (SNPs) and defined how this candidate gene influences infection phenotypes, notably progression to active TB, as well as the extent of pulmonary inflammation during active disease.

## Materials and Methods

### Genotyping

NCBI SNP database was used to select the CARD9 SNPs previously associated with chronic inflammation (rs4077515 [9, 10, 13, 14]; rs10870077 [15, 16]; rs10781499 [15, 17, 18]). rs4077515, a nonsynonymous SNP located in codon 2 of *CARD9* gene, leads to an amino acid substitution at position 12 (p.Ser12Asn) and appears”benign” for protein function [19]. The rs10781499 is a synonymous SNP located in the third exon of the gene with undefined functional effects [20], while rs10870077 is an intronic variant that might modify *CARD9* expression [16]. DNA was isolated and purified from blood samples collected on EDTA from 716 Caucasian subjects (342 patients diagnosed with active pulmonary TB and 374 healthy controls). The Ethics Committee of University of Medicine and Pharmacy of Craiova, Romania, approved this study (Ethical approval No.03/07.02.2011) and all subjects provided written informed consent. All subjects were HIV- and type II diabetes-negative. Clinical characteristics of subjects included in the study, both TB and healthy controls, are presented in Table 1. Demographic data, total and differential leukocyte counts, erythrocyte sedimentation rate (ESR) and body-mass index (BMI) were recorded at TB diagnosis, prior to initiation of the chemotherapy. The algorithm employed to establish the TB diagnosis followed the recommendations of the WHO [21]. Patients with clinical signs indicative of active disease, TB suggestive chest radiographic findings and Mtb-positive sputum samples were classified as TB cases.

**Table 1:** Demographic and clinical features of study subjects.

Group	Gender ratio (F/M)	Age (y)	BMI	WBC count (cells/mm^3^)	Neutrophil count (cells/mm^3^)	Baseline ESR
**Active TB** (n = 342)	62 / 280	50.23±14.05	21.20±3.99	8.84±3.46	6.2±3.29	45±43.72
**Healthy controls** (n = 374)	156 / 218	59.38±13.82	25.11±4.6	6.8±1.85	4.43±1.5	30±22.03

BMI, Body mass index; WBC, White blood cell; ESR, Erythrocyte sedimentation rate. Values are mean±SD.

Genomic DNA was prepared from peripheral blood leukocytes according to the standard protocol of Wizard® Genomic DNA Purification Kit (*Promega*, *Madison*, *WI)*. Genotyping for the *CARD9* polymorphisms was performed with the predesigned TaqMan single nucleotide assays (C_25956930_20, C_32127979_20 and C_25957125_10) on the ViiA7 Real-Time Polymerase Chain Reaction system (all from Applied Biosystems, Foster City, CA).

### Plasma cytokine and chemokine measurements

Cytokine and chemokine concentrations were measured in all available plasma samples. Specimens were collected prior to chemotherapy against TB, upon confirmation of active disease. Abundances of immune mediators were determine by multiplex magnetic bead immunoassay (Luminex assay, R&D Systems), with a Bio Plex microbead analyzer (Bio-Plex^®^ 200 Systems, Bio-Rad Laboratories, Inc.) according to the manufacturer’s protocol.

### Statistical analyses

Statistical analyses were performed with the R programming language, version 3.1.1. Association between haplotypes and disease status (TB vs. non TB) was tested using Fisher's exact test. For machine learning, we used the random forest algorithm as implemented in the randomForest R package [22]. Associations between haplotypes and continuous variables (quantitative locus trait analysis) were tested using analysis of variance. Binomial logistic regression was used to test the effect of continuous variables on categorical outcomes (BMI and age as predictors for the TB vs. control outcome). Local linear polynomial regression was performed using the loess function in R. PCA was performed with the *prcomp* function in R and visualized with the pca3d package [23]. Multiple correspondence analysis for calculation of the disease severity score was carried out with the *mca* function from the MASS R package [24]. For post-hoc ANOVA tests, the Tukey Honest Significance Differences test was used.

## Results and Discussion

Recent genome-wide association studies and meta-analyses indicate that selected *CARD9* SNPs result in predisposition to chronic inflammatory disorders [8–10, 25]. Based on our previous study in murine TB [4] and considering that TB is characterized by non-resolved inflammation, we hypothesized that similar genetic variants could influence progression of latent to active TB. To test our hypothesis, genotype distributions of three *CARD9* polymorphisms, -201C/T (rs4077515), -311G/C (rs10870077), and -126C/T (rs10781499) were tested in a Caucasian cohort of Romanian ethnicity. Vaccination is mandatory in this TB-endemic region and thus we anticipated that subjects enrolled in our study had been immunized as infants with the vaccine *M*. *bovis* Bacille Calmette-Guérin (BCG). The analyzed SNPs showed linkage disequilibrium and haplotypes were equally distributed in both TB and healthy groups (Table 2). Our findings are in line with reports nullifying a link between *CARD9* rs4077515 genotypes and susceptibility to candidemia and recurrent vulvovaginal candidiasis [13, 19]. However, a lack of association between *CARD9* variants and active TB diverges from associations reported for several chronic inflammatory diseases, including Crohn’s disease [8, 10, 16] and ankylosing spondylitis [15]. Despite the chronic feature of adult pulmonary TB, preexisting anti-mycobacterial immunity in the population studied could mask effects of *CARD9* polymorphisms on TB progression.

**Table 2:** *CARD9* rs4077515, rs10870077, rs10781499 major haplotypes in active TB patients and healthy controls.

Haplotype	Group	
	Healthy controls	Active TB
CC.CC.GG	161	140
CT.CC.GG	2	7
CT.CG.AG	166	161
CT.GG.AG	1	0
TT.CG.AG	1	1
TT.GG.AA	42	33

p-value (Fisher's Test) = 0.3671.

**Fig 1:**
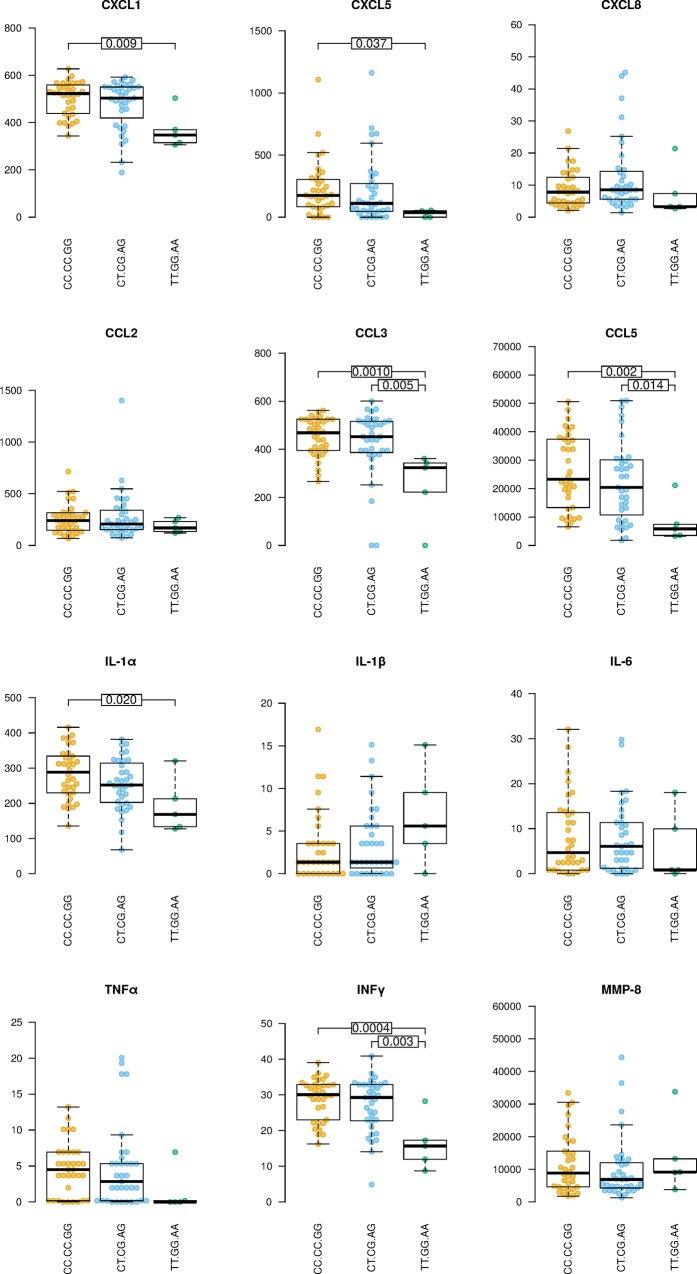
*CARD9* genetic variants affect abundances of selected chemokines and cytokines in active TB. Abundances (pg/ml) of selected soluble immune mediators for the three major haplotypes (rs4077515, rs10870077, rs10781499) analyzed. Each point corresponds to a single sample. Horizontal bars show the p-value in post-hoc Tukey Honest Significance Differences test, calculated after an initial ANOVA on log-transformed data.

**Fig 2:**
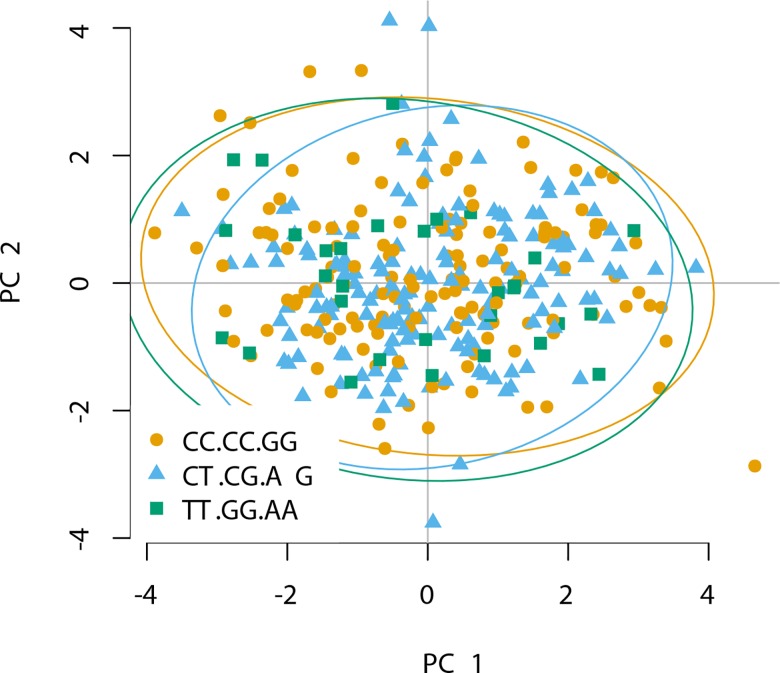
Association between *CARD9* haplotypes and clinical parameters in TB patients. PCA of samples from TB patients applied to clinical covariates (age, BMI, WBC, neutrophils, lymphocytes and ESR) and colored by haplotype.

We further investigated whether distinct *CARD9* variants influence inflammatory responses in active TB. Plasma abundances of TB-relevant cytokines and chemokines were determined in a sub-cohort of TB cases. Individuals carrying the TT.GG.AA haplotype produced significantly lower levels of IFN-ɣ, CCL3, and CCL5 compared with the other two major haplotypes (Fig 1). IFN-ɣ is critical for defense against TB [26], yet high concentrations could indicate more severe pathology [27]. Because the CC chemokines orchestrate the dynamics of leukocytes, and reduced abundance reflects limited inflammation, we therefore expected an effect of the genotypes on clinical manifestation of TB. This presumption was not supported by principal component analysis (PCA) integrating clinical covariates (Fig 2). The clinical covariates included BMI and parameters allowing evaluation of emergency hematopoiesis, such as whole blood cell (WBC) counts and frequencies of neutrophils and lymphocytes, as well as ESR, a marker of systemic inflammation. No correlation between the major *CARD9* haplotypes and principal components was observed, thus revealing similar systemic inflammation irrespective of the gene variant. These findings could be due to variable expression of the *CARD9* gene in distinct haplotypes. Alternatively, tested SNPs regulate expression quantitative trait loci (eQTL) in *trans*. A *cis* eQTL effect of the tested SNPs is unlikely, as genes neighboring *CARD9* are not directly associated with immune functions.

**Fig 3:**
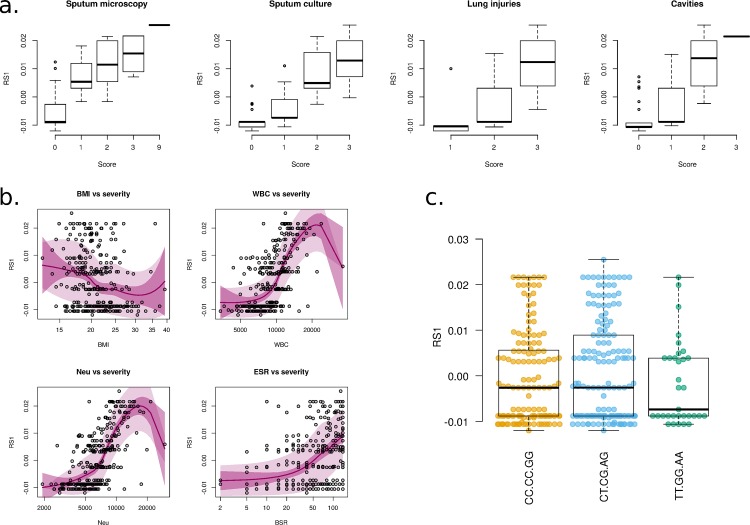
Severity of pulmonary TB is not linked to *CARD9* haplotypes. **(a)** Disease severity score RS1 calculated using multifactor correspondence analysis (MCA) based on the clinical scores obtained from parameters monitored during sputum microscopy and chest X-ray; **(b)** Local polynomial regression on four clinical covariates (BMI, WBC, neutrophils and ESR) and severity score RS1 derived from MCA for samples from TB patients. Each point corresponds to a single sample. Light colored area, 95% prediction confidence interval; dark colored area, 95% estimation confidence interval; (**c**) TB severity score among patients carrying various *CARD9* haplotypes.

In order to assess how distinct *CARD9* SNPs affect disease severity we first elaborated a severity score (RS1). RS1 served as a disease proxy (Fig 3A) and was built on sputum microbiology and chest X-ray scores (Table 3). RS1 showed a direct correlation with leukocyte count, including WB and neutrophil counts, and ESR values, and furthermore it was inversely correlated with the BMI (Fig 3B). Indeed, neutrophilia represents a marker of poor prognosis in TB patients [28] and heightened neutrophil frequencies correlate with severity of pulmonary TB [29]. These observations support our newly defined disease proxy parameter. However, patients carrying various *CARD9* haplotypes presented similar disease severity scores (Fig 3C). Although *Card9* mutant mice [4] develop severe inflammation during TB, in the case of humans, inflammatory mediators and disease severity did not differ profoundly in patients carrying distinct *CARD9* variants. The disparity between mouse and man is unlikely to rely on the variability of outcomes measured. In both mice and humans we measured several inflammatory parameters to monitor systemic (sera/plasma levels of cytokines) and lung inflammation. Indeed, in humans we employed RS1 to overcome inaccessibility of the lung tissue and assess the lung involvement whereas in mice we evaluated lung changes by histopathologic examination of the tissue. RS1 was built on lung radiographic changes, correlated very well with systemic inflammation markers, which increase upon exacerbated lung damage [29], and thus emerged as a valuable surrogate of pathology, as used in mice.

**Table 3:** Sputum microbiology and chest X-ray scoring.

Grading	Sputum Score		Chest X-ray Score**	
	Sputum Microscopy	Sputum Culture	Lung injuries	Cavity score
**0**	No Mtb* detected	No colonies	No lesions	No cavities
**1**	10–99 Mtb/on 100 fields	30–100 colonies	Minimal lung damage (infiltrative lesions with Φ <3 cm)	Φ = 2–4 cm
**2**	1–10 Mtb/field	>100 single colonies	Mild lesions (infiltrative lesions 3 ≤ Φ ≤ hemithorax; disseminated lesions ≤ 1/2 of hemithorax area or 1/3 of hemithorax volume; dense and confluent opacities + cavities)	Φ = 4–8 cm
**3**	>10 Mtb/field	Confluent growth	Severe/extended lesions (exceeding the above criteria)	Φ ˃ 8 cm

*Mtb, *Mycobacterium tuberculosis*

**Blind assessment of lung chest X-ray images by two different clinicians.

The phenotype reported in mice largely relies on expression of *Card9* in neutrophils for IL-10 release, whereas human neutrophils are unable to produce this regulatory cytokine due to an inactive chromatin configuration at the *IL10* locus [30]. This difference could explain discrepancies observed between mice and humans regarding *CARD9* control of inflammation. Moreover, the observed discrepancy between *Card9* knock-out mice and severity of TB in humans can rely on loss-of-function in the murine system, while in humans the SNPs could result in lower abundance of or modifications in the CARD9 protein structure leading to signaling defects. We did not determine the expression level of the *CARD9* gene or abundances of the encoded protein in blood specimens of variable haplotypes. Of note, the *CARD9* gene is under significant *cis* regulation [15] and presumably single SNPs minimally affect protein structure.

In this study we provide evidence that *CARD9* genetic variants in rs4077515, rs10781499 and rs10870077 do not affect progression to active TB in a cohort from a TB-endemic region with an ongoing BCG vaccination program. In mice, vaccination has been found to protect the *Card9*-deficient animals against lethality due to TB [4]. Genetic studies in BCG-naive humans are required before conclusions can be made on the development of active TB, irrespective of the *CARD9* haplotypes. Moreover, our study was undertaken in a single cohort. Investigations in additional populations and larger cohorts will need to overcome such limitations. We have guided our study by reports centered on chronic inflammation, and tested selected SNPs. As the effects of additional *CARD9* SNPs on progression to active TB cannot be excluded, a fine-mapping of the *CARD9* gene could help elucidate whether other gene variants imprint on susceptibility to TB in humans.

While the selected *CARD9* SNPs predispose to chronic inflammation in multifactorial inflammatory diseases [10, 14, 25, 31], their roles in human infections driven by specific microbes, such as Mtb, as shown here, and *Candida* spp. [13, 19], appears limited. Nonetheless, more than 15 missense and nonsense *CARD9* point mutations (Q295X; Q289X; R70W; R35Q or R101L are some of the rare variants identified) have been associated with development of fungal diseases [32–36]. Such mutations result either in synthesis of a nonfunctional protein or lack of the CARD9 protein. In contrast to loss-of-function mutations, *CARD9* SNPs including rs4077515, leave cytokine production unchanged in leukocytes stimulated with fungi. The SNP thus minimally affects the function of the adaptor protein and is uncoupled from susceptibility to candidemia [19]. Unlike previous reports in inflammatory polyfactorial diseases, selected SNPs do not affect the course of TB or fungal infection. Additional studies investigating supplementary *CARD9* SNPs and effects of *CARD9* variants in unrelated infections will permit insights into divergent roles of this adaptor molecule in chronic inflammation of microbial and non-microbial origin.
