# Correction: The *CARD9* Polymorphisms rs4077515, rs10870077 and rs10781499 Are Uncoupled from Susceptibility to and Severity of Pulmonary Tuberculosis

**DOI:** 10.1371/journal.pone.0165853

**Published:** 2016-10-26

**Authors:** Ioana Streata, January Weiner, Marco Iannaccone, Gayle McEwen, Marius Sorin Ciontea, Marian Olaru, Rosanna Capparelli, Mihai Ioana, Stefan H. E. Kaufmann, Anca Dorhoi

The third author's name is spelled incorrectly. The correct name is: Marco Iannaccone. The correct citation is: Streata I, Weiner J 3rd, Iannaccone M, McEwen G, Ciontea MS, Olaru M, et al. (2016) The *CARD9* Polymorphisms rs4077515, rs10870077 and rs10781499 Are Uncoupled from Susceptibility to and Severity of Pulmonary Tuberculosis. PLoS ONE 11(9): e0163662. doi:10.1371/journal.pone.0163662
